# Quantifying exposure of amphibian species to heat waves, cold spells, and droughts

**DOI:** 10.1111/cobi.70074

**Published:** 2025-05-31

**Authors:** Evan Twomey, Francisco Sylvester, Jonas Jourdan, Henner Hollert, Lisa M. Schulte

**Affiliations:** ^1^ Department of Wildlife, Zoo‐Animal‐Biology and Systematics, Faculty of Biological Sciences Goethe University Frankfurt Frankfurt am Main Germany; ^2^ Department of Evolutionary Ecology and Environmental Toxicology, Faculty of Biological Sciences Goethe University Frankfurt Frankfurt am Main Germany; ^3^ Department of Aquatic Ecotoxicology Goethe University Frankfurt am Main Frankfurt am Main Germany; ^4^ Department of Environmental Media Related Ecotoxicology Fraunhofer Institute for Molecular Biology and Applied Ecology IME Schmallenberg Germany

**Keywords:** amphibian declines, climate change, droughts, extreme events, heat waves, cambio climático, declinación de anfibios, eventos extremos, olas de calor, sequías

## Abstract

Globally, amphibians face severe threats, such as climate change and associated extreme events. Our goal was to quantify global amphibian exposure to 3 classes of extreme events: heat waves, cold spells, and droughts. We used the MERRA‐2 extreme climate events data and the standardized precipitation–evapotranspiration index database to investigate where these events have increased over the last 40 years. We used the International Union for Conservation of Nature (IUCN) database of global amphibian distributions (7202 species) to calculate the level of exposure to extreme events for each species, classifying species as exposed if their distribution had ≥50% overlap with areas experiencing substantial increases of extreme events. To assess whether exposure is associated with amphibian declines, we used logistic regression to analyze the relationship between extreme event exposure and status changes on the IUCN Red List. Heat waves and droughts increased notably in Amazonia, Madagascar, and Europe. Among the 3 classes of events, exposure was highest to heat waves (40% of species exposed), followed by droughts (16% exposed). Exposure to different event classes was uneven with respect to geography and taxonomy. Some areas (e.g., Amazonia, Madagascar) and families (e.g., Mantellidae, Rhinodermatidae) had nearly 100% of constituent species classified as exposed to at least one event class. Exposure to heat waves (odds ratio 1.8) and droughts (odds ratio 1.7) was associated with status deteriorations since 2004. Our findings provide insight into amphibian biodiversity hotspots and taxonomic groups that may be particularly susceptible to extreme climate events, suggesting that these events play a causative role in ongoing declines. Understanding the aspects of species biology that influence susceptibility to extreme events, as well as interactions with other factors (e.g., disease), will be important for understanding the role of climate change in driving amphibian declines.

## Abstract (es)

Cuantificación de la exposición de las especies de anfibios a las olas de calor, frentes fríos y sequías

**Resumen:**

Los anfibios se enfrentan a graves amenazas en todo el mundo, como el cambio climático y los eventos extremos asociados. Nuestro objetivo fue cuantificar la exposición global de los anfibios a tres clases de eventos extremos: olas de calor, frentes fríos, y sequías. Utilizamos los datos sobre fenómenos climáticos extremos de MERRA‐2 y la base de datos del índice normalizado de precipitación‐evapotranspiración para investigar dónde han aumentado estos fenómenos en los últimos 40 años. Utilizamos la base de datos de la Unión Internacional para la Conservación de la Naturaleza (UICN) sobre la distribución mundial de los anfibios (7,202 especies) para calcular el nivel de exposición a eventos extremos de cada especie, con las especies con distribución solapada en ≥50% con zonas bajo aumentos sustanciales de eventos extremos clasificadas como expuestas. Para evaluar si la exposición está asociada al declive de los anfibios, utilizamos la regresión logística para analizar la relación entre la exposición a fenómenos extremos y los cambios de estado en la Lista Roja de la UICN. Las olas de calor y las sequías aumentaron notablemente en la Amazonia, Madagascar y Europa. Entre las tres clases de eventos, la exposición más elevada fue a las olas de calor (40% de las especies expuestas), seguidas de las sequías (16% expuestas). La exposición a las distintas clases de eventos fue desigual con respecto a la geografía y la taxonomía. En algunas zonas (por ejemplo, Amazonia y Madagascar) y familias (por ejemplo, Mantellidae y Rhinodermatidae), casi el 100% de las especies constituyentes se clasificaron como expuestas al menos a una clase de evento. La exposición a olas de calor (razón de probabilidades: 1.8) y sequías (razón de probabilidades: 1.7) se asoció con deterioros del estado desde 2004. Nuestros hallazgos proporcionan información sobre los puntos calientes de la biodiversidad de anfibios y los grupos taxonómicos que pueden ser particularmente susceptibles a los eventos climáticos extremos, lo que sugiere que estos eventos juegan un papel causal en la disminución en curso. El entendimiento de los aspectos de la biología de las especies que influyen en la susceptibilidad a los fenómenos extremos, así como las interacciones con otros factores (por ejemplo, las enfermedades), será importante para entender el papel del cambio climático en la declinación de los anfibios.

## INTRODUCTION

Amphibians, with 8687 species worldwide (Frost, 2023), are the most threatened vertebrate class on the planet (Luedtke et al., 2023). According to the most recent assessment by the International Union for Conservation of Nature (IUCN), 41% of amphibian species are currently threatened with extinction, a number that has increased in recent years (e.g., 33% in 2006; Luedtke et al., 2023; Stuart et al., 2008). These declines are due to a variety of factors, with habitat loss being among the most important (Ficetola et al., 2015; Gallant et al., 2007). Additionally, disease, pollution, and climate change also contribute to amphibian declines (Hayes et al., 2010; Scheele et al., 2019; Winter et al., 2016).

Climate change poses an increasingly severe threat to amphibians (Corn, 2005; D'Amen & Bombi, 2009; Li et al., 2013; Luedtke et al., 2023). Several studies make predictions regarding the effects of general climatic changes (e.g., temperature shifts) on amphibians (Miller et al., 2018; Schivo et al., 2019). However, extreme climatic events, such as heat waves, droughts, and floods, are also part of climate change and are becoming more frequent and more severe (Coumou & Rahmstorf, 2012; IPCC, 2014; Swain et al., 2020). Given the specific requirements of many amphibian species, usually being dependent on either fresh water, wet terrestrial habitats, or both, as well as their strong dependence on humidity and temperature, these extreme events can have profound impacts on amphibian populations. Such impacts include direct mortality of adults, juveniles, or larvae (Burrowes et al., 2004; Cayuela et al., 2016; Heyer et al., 1988), increasing susceptibility to disease (Adams et al., 2017; Moura‐Campos et al., 2021; Rohr & Raffel, 2010), and disruption of breeding cycles by accelerating the drying of temporary pools (Daszak et al., 2005; Walls et al., 2013).

The specific impacts of extreme events are often unknown and may vary depending on intensity, timing, and life‐history traits, such as the reproduction strategy of the species in question (Li et al., 2013; Maxwell et al., 2019; Walls et al., 2013). However, one key factor thought to influence the severity of an event's effects is the extent to which a system (e.g., species) is exposed to climatic variations (IPCC, 2001). This level of exposure can be defined spatially, for example, by determining the amount of spatial overlap between the distribution of a species and given extreme event, where greater exposure is expected to increase the probability that a species will be negatively affected (Ameca y Juárez et al., 2013). Thus, one way to identify the potential impacts of climate change on amphibians is to quantify exposure of amphibian species to different kinds of extreme events. Such an approach allows for the identification of geographic areas (e.g., biodiversity hotspots) or taxonomic units (e.g., orders, families) that are particularly exposed and thus may be at risk for experiencing declines. However, such an approach based on past or predicted exposure may fail to account for future scenarios where extreme events may occur in unforeseen locations.

Among extreme events, temperature and precipitation anomalies affect amphibians especially severely and have been implicated in amphibian declines (Li et al., 2013; Maxwell et al., 2019; Rohr & Raffel, 2010). Impacts include direct lethal effects, such as the desiccation of postmetamorphic amphibians and premature drying of aquatic habitats leading to larval mortality and declines (Cayuela et al., 2016; Daszak et al., 2005; McMenamin et al., 2008). In addition, hot and dry periods can reduce water depth, increasing embryonic UVB exposure, which can exacerbate the effects of pathogens, such as *Saprolegnia* (water mold), trematodes, and the fungal disease *Batrachochytrium dendrobatidis* (Bd) (Burrowes et al., 2004; Kiesecker, 2011; Moura‐Campos et al., 2021). However, heat waves need not necessarily be associated with dry periods to negatively affect amphibians. Experimentally induced 6‐day heat waves reduce survival and growth of amphibian larvae and may also cause sex reversal (Ujszegi et al., 2022). Temperature extremes in natural settings can also exceed the thermal limits of amphibian larvae, particularly those occurring in subtropical habitats with low canopy cover (Duarte et al., 2012).

Periods of abnormally cold weather (cold spells) may also negatively affect amphibians, most notably through interactions with pathogens, such as chytrid fungi. Growth and virulence of Bd are strongly influenced by temperature; outbreaks are often observed during cool seasons or in species occurring at higher elevations (Doddington et al., 2013; Kinney et al., 2011; Kriger & Hero, 2007; Raffel et al., 2013; Retallick et al., 2004; Scheele et al., 2019; Walker et al., 2010). This may be due to an increase in zoospore release and persistence, reduction in immune response, shifts toward the thermal optimum of Bd, or combinations of these factors (Doddington et al., 2013; Fisher et al., 2021). Similar results of cold‐enhanced virulence have been found with the salamander chytrid fungus, *Batrachochytrium salamandrivorans* (Carter et al., 2021). Furthermore, shifts in temperature, particularly in the direction of warm to cold, can increase Bd virulence (Raffel et al., 2013). Overall, the data suggest cold temperatures in general and shifts to cold temperatures, such as experienced during a cold spell, can exert negative effects on amphibians, particularly through increased disease emergence (Bradley et al., 2019; Campbell et al., 2019; Drew et al., 2006; Heyer et al., 1988).

We sought to quantify the exposure of global amphibian diversity to extreme events in order to identify taxonomic groups and regions that may be especially at risk. We focused on 3 classes of extreme events—heat waves, droughts, and cold spells—and used MERRA‐2 and standardized precipitation–evapotranspiration index (SPEI) climate data over the past 4 decades to identify areas where the frequency of extreme events has increased since 1980. We used a global data set of amphibian distributions to identify geographic areas where high numbers of amphibian species are classified as significantly exposed to extreme events. Finally, we used a recently published data set (Luedtke et al., 2023) to test whether exposure to extreme events is associated with deteriorating status of species on the IUCN Red List.

## METHODS

### Data

**FIGURE 1:**
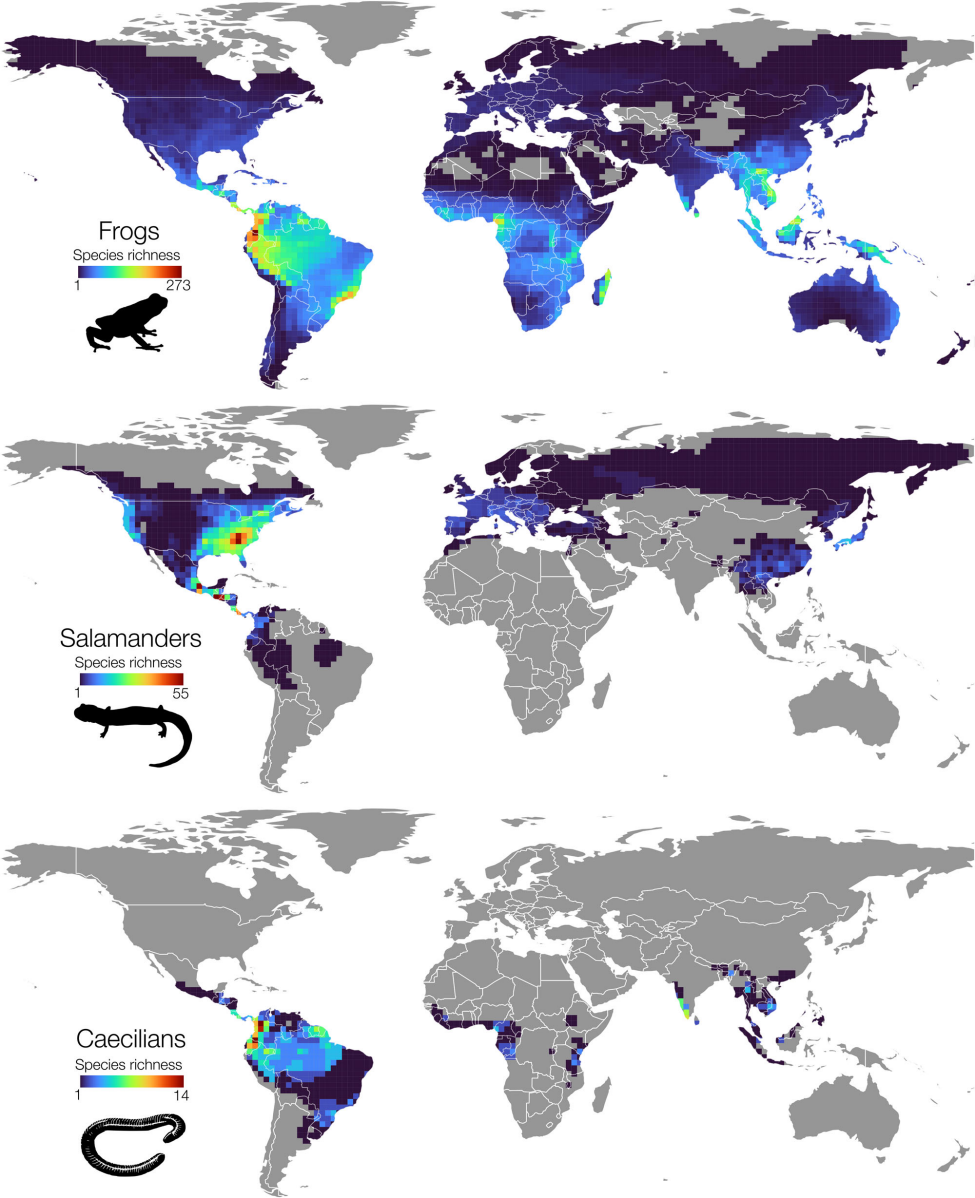
Global species richness of 3 amphibian orders: frogs (Anura), salamanders (Caudata), and caecilians (Gymnophiona) (gray, no species present). Species counts are summed per 2° grid cell. Species distribution data are from the International Union for Conservation of Nature Red List 2018‐2.

Amphibian species distribution maps were obtained from the IUCN Red List 2018‐2 (accessed 24 November 2021 from https://www.iucnredlist.org/resources/spatial‐data‐download) (IUCN, 2021). This data set consists of shapefiles for all species of amphibians, which at the time of access was 6339 species of Anura, 695 species of Caudata, and 168 species of Gymnophiona, for a total of 7202 amphibian species. These range maps delimit outer range boundaries of species rather than isolating grid cells where the species actually occurs and are thus relatively coarse representations of a species’ distribution. We used this data set to calculate global species richness for each order by summing species counts per 2° global grid cell (Figure 1). Although a smaller choice of grid cell may reveal finer‐scale patterns, it also underestimates species richness in poorly sampled areas. For example, the high diversity of Neotropical plethodontid salamanders, many of which are known from only a single locality, was not as apparent at a 0.5° or 1° resolution compared with 2° because each of the finer cells only captured a small fraction of the much larger regional richness. This and all other spatial analyses were done in QGIS 3.22.4 (QGIS Development Team, 2021).

We followed the existing climate‐based definitions of extreme events rather than definitions based on expected biological effects, which could be evenly applied to all species in the analysis (see below for definitions). Briefly, this includes the counts of 6‐day heat waves, 6‐day cold spells, and 3‐month severe droughts based on an index accounting for both precipitation and temperature. Heat wave and cold spell extreme event data were derived from the MERRA‐2 data from NASA (Gelaro et al., 2017). Specifically, we accessed the single‐level, monthly extremes detection indices V2 (M2SMNXEDI) via the Giovanni portal (Collow et al., 2021; date of access 25 March 2022 from https://giovanni.gsfc.nasa.gov/giovanni/). This data set covers the period 1980 to present and consists of indices used to identify extreme weather events associated with temperature. Because heat waves and cold spells are defined by anomalous temperatures over a short period (e.g., several consecutive days), we analyzed metrics that capture both these essential features. For heat waves, we analyzed the monthly count of heat wave events, defined as 6 consecutive days with a temperature exceeding the 90th percentile for that grid cell. For cold spells, we used the monthly count of cold spell events, defined as 6 consecutive days with a temperature below the 10th percentile (Alexander, 2016).

For droughts, we used the SPEI (SPEIbase, version 2.6) (Vicente‐Serrano et al., 2010) (accessed 24 March 2022 from https://digital.csic.es/handle/10261/202305). Unlike drought indices based solely on precipitation (such as the SPI), SPEI includes temperature data to account for increased water stress due to evapotranspiration, which might especially affect amphibians due to increased desiccation risk for adults and juveniles and premature drying or inadequate filling of breeding pools, such as vernal pools, wetlands, and phytotelmata. Because droughts are defined by a lack of water inputs over a period of time, the time scale over which drought indices are calculated is a key consideration. We initially analyzed 3‐month and 12‐month aggregation periods but found that the two gave similar results in terms of identifying areas of increased drought frequency. Therefore, we focused only on the 3‐month SPEI data (SPEI3) because this corresponds to the length of the breeding season and larval period for many temperate species. The global SPEI database covers the period 1901 to the present. For our purposes and for consistency with the heat wave and cold spell data (which starts in 1980), we only used SPEI data from 1980 to the present.

With the above data sets, we were interested in determining whether the frequency of extreme events increased over time and, if so, in which regions of the world. For example, if extreme events are increasing due to climate change, this should lead to an increase in the frequency of extreme events in recent years compared with an earlier period (Coumou & Rahmstorf, 2012). To address this, for each of the three extreme event classes, we compared recent data (2010–2019) with data from a baseline period (1980–1989). For heat wave and cold spells, we summed the monthly count of events over the period 2010–2019 and subtracted the count of events during the baseline period, 1980–1989. For droughts, positive SPEI values indicate wetter‐than‐normal conditions, and negative values indicate drier‐than‐normal conditions. Thus, we defined a drought event as a monthly SPEI3 <−1.5, which represents severe drought (Paulo & Pereira, 2006).

To be able to express our results as the number of species at risk per any given area or taxonomic group, we converted monthly SPEI3 rasters to binary rasters by retaining cells <−1.5 and summed these rasters over the two decadal periods, resulting in a count of SPEI3 −1.5 drought events for each period. As with the counts of heat wave and cold spell events, we subtracted the counts of drought events during the baseline period from the counts during 2010–2019 to identify where the frequency of extreme events increased over time.

**FIGURE 2:**
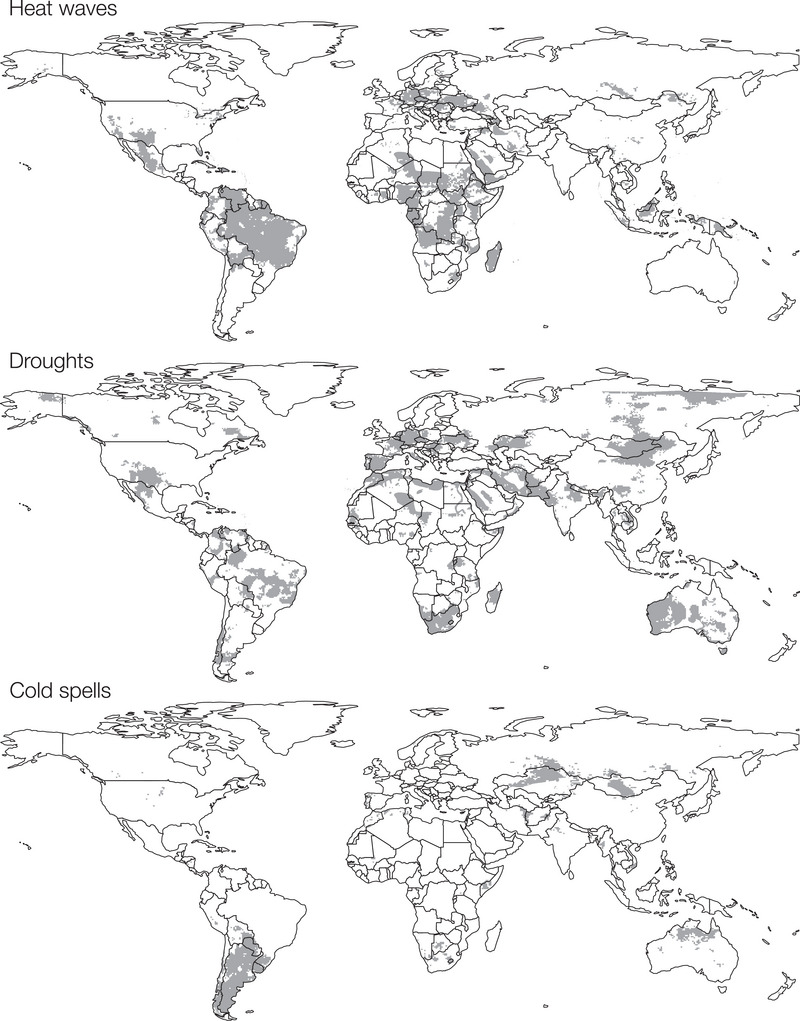
Extreme heat, drought, and cold event layers used in the analyses of exposure of amphibian species to extreme events. For heat waves and droughts, shaded cells indicate the 80th percentile of the event count difference in 2010–2019 minus the baseline period of 1980–1989. For heat waves, this corresponds to values with ≥32 heat wave events (recent minus baseline) and for droughts ≥10 events (recent minus baseline). For cold spells, given the overall trend toward fewer cold spell events over time, the threshold value for the layer corresponds to ≥0, that is, all cells where there was an increase in cold spell events since the baseline period (corresponding to the 94th percentile). Extreme events are defined in “METHODS.”

As a final step, to obtain simple extreme‐event layers, we converted the difference‐in‐counts rasters to binary rasters (Figure 2). For heat wave and drought counts, this was done at an 80th percentile cutoff (see results for specific values used). For cold spells, given that the 80th percentile included negative values (i.e., areas where cold spells decreased over time), we instead retained only positive values in the binary raster (i.e., including all grid cells where the count of cold spells increased over time). Our logic behind this procedure was to capture the tails of the distribution of the changes in event frequency. Focusing on the more extreme values of event increase allowed us to focus on the most severely affected regions and taxonomic groups. The resulting extreme event layers at different percentile thresholds are shown in Appendix S1. These calculations were done with a clipping mask representing the global distribution of the class Amphibia; thus, they excluded areas where there was no reported amphibian occurrence. To quantify the magnitude of exposure to extreme events, we used the difference‐in‐counts raster to count the number of excess extreme events per grid cell per decade within the range of each species. This was done with the zonal statistics function in QGIS. All results regarding extreme event exposure are given in Appendix S2.

### Quantifying exposure to extreme events

To classify amphibian species as exposed (or not) to each class of extreme event, we used overlap analysis in QGIS to calculate the total area of distribution overlap per species with the extreme event layer. Given that many species have distributions represented by more than one polygon (e.g., species with disjunct populations), total overlap areas and total distribution areas were summed by species and used to calculate the overall overlap proportion for a species. This was done at the native resolution of the input layers (0.5 × 0.625° for MERRA‐2 data, 0.5° × 0.5° for SPEI, and vector shapefiles for IUCN distribution data). We classified species as exposed when the percentage of their distribution overlapping with the extreme event layer was >50%, a relatively conservative cutoff (e.g., Ameca y Juarez et al., 2013). After classification, we tallied species counts by taxonomic group (order and family) to count numbers and percentages of exposed species in each group.

To derive counts of exposed species over geographical areas, we filtered the amphibian distribution shape files by exposure status, retaining only species classified as exposed. We then used the “join attributes by location (summary)” function in QGIS to count the number of species overlapping with the geographic layer. For the latter, we used a global 2° grid, which provided counts of exposed species per 2° cell, and a shape file of political boundaries, which provided counts of exposed species per country. Using the counts of exposed species per 2° grid cell, we also divided this by the total number of species in that grid cell to derive the relative proportion of exposed species in relation to local species richness.

### Extreme event exposure and changes in IUCN Red List status

Luedtke et al. (2023) reported the findings of the second Global Amphibian Assessment (GAA2), a systematic assessment of extinction risk for all the world's amphibians. First completed for amphibians in 1980, this assessment was updated in 2004 (GAA1) and again in 2022 (GAA2), allowing for an assessment of deteriorations (or improvements) in the status of species over time.

To investigate whether exposure to extreme events was related to changes in conservation status, we accessed the data from Luedtke et al. (2023) (available at https://www.iucnredlist.org/resources/data‐repository). Although these classifications provide a relatively coarse metric of species responses and may fail to account for short‐term, localized effects of extreme events, they have the advantage of capturing species‐level responses across a large number of species at a global scale. We used multinomial logistic regression to test whether extreme event exposure (continuous from 0 to 1, expressed as the proportion overlap between the range of a species and the binary extreme event layer) was associated with a change in conservation status (categorical outcome). These outcomes included no change in status, status deterioration (uplisted), or status improvement (downlisted). Two analyses were run, corresponding to the different periods of the GAA1 and GAA2 (i.e., comparing changes in status between the 1980 assessment and the GAA1 in 2004 and changes in status between the GAA1 and GAA2 in 2022). We performed this analysis with the multinom_reg function with exponentiate = TRUE (to return odds ratios) from the tidymodels R package (Kuhn & Wickham, 2020) in R 4.1.2 (R Core Team, 2021).

## RESULTS

### Heat waves

During the baseline period (1980–1989), the global median count of heat wave events per grid cell was 66. During 2010–2019, this increased to a median of 80 events per cell; 79.7% of grid cells showed an increase in heat wave count and a median increase of 14 heat wave events per grid cell. For conversion to a binary raster for use in downstream analyses, we used a threshold value of 32, corresponding to the 80th percentile of heat wave event increase (i.e., including only those grid cells experiencing an increase of 32 or more heat wave events since the baseline period). The greatest increase in the number of heat waves occurred throughout most of Amazonia, Madagascar, central Africa, most of Borneo, southwestern United States, Mexico, and much of continental Europe (Figure 2).

Classifying amphibian species as exposed when >50% of their distribution overlapped with the heat wave layer, we found that 43% of Anura (frogs) and 41% of Gymnophiona (caecilians) were exposed to heat waves, given that much of the diversity of these 2 orders is concentrated in tropical regions, such as Amazonia and Madagascar (Figure 1). In contrast, only 14% of salamanders were classified as exposed to heat waves because the bulk of their diversity is concentrated in the eastern United States and tropical Mesoamerica (Figure 1), where heat waves did not appear to have increased to the same degree (Figure 2).

**FIGURE 3:**
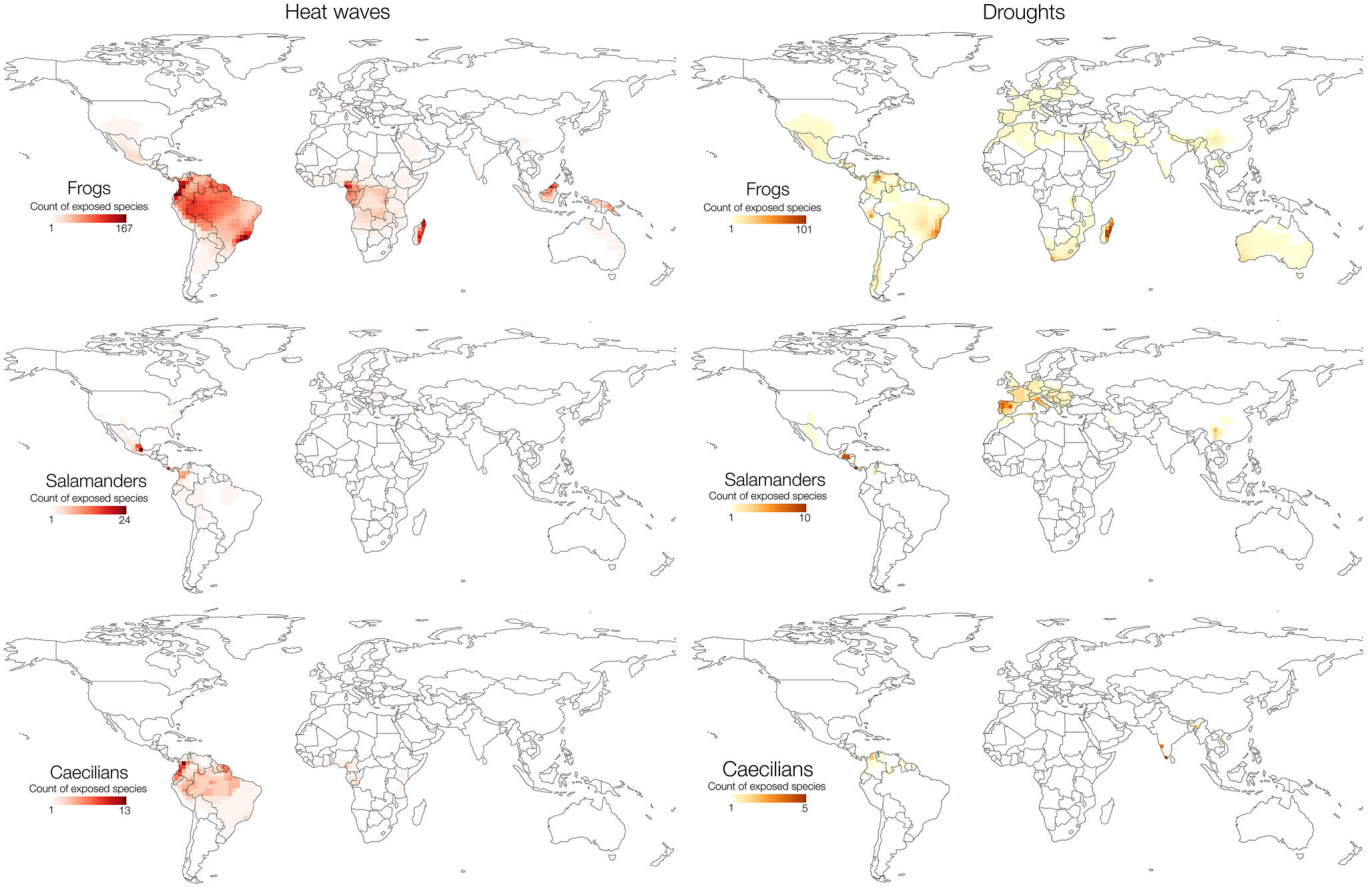
Number of amphibian species exposed to heat waves and droughts per global 2° grid cell. A species is exposed if >50% of its distribution overlaps with the extreme event layer (see Figure 2).

South America had the highest numbers of frogs exposed to heat waves, particularly in the Chocó, Atlantic Forest of Brazil and throughout most of the Amazon basin (Figure 3). Brazil had the highest number of heat‐wave‐exposed frog species (613), followed by Colombia (541), Venezuela (333), Madagascar (293), and Ecuador (284). Exposed salamanders occurred mostly in Mexico (48 species), Colombia (23), and Panama (15).

When expressed as a proportion (number of exposed species in a given grid cell divided by the total species in that grid cell), our analyses revealed that throughout most of the Amazon, Madagascar, and central Africa, nearly all amphibian species in those areas were classified as heat wave exposed (Figure 5). Among other species‐rich areas, Mexico, Cuba, Hispaniola, Borneo, and New Guinea also had high proportions of heat‐wave‐exposed amphibian species (Figure 5).

Among frog families, Mantellidae (endemic to Madagascar) had the highest exposure to heat waves; 210 of its 215 species (98%) were classified as heat wave exposed. Aromobatidae, distributed throughout much of South America, also contained a high number of heat‐wave‐exposed species (91 of 113 species, 81%). Other highly exposed families included Petropedetidae (tropical Africa, 9 of 12 species), Hylodidae (Atlantic Forest region of Brazil, 29 of 41 species, 71%), and Leptodactylidae (Central and South America, 120 of 180 species, 67%). Five caecilian families had greater than half their species classified as heat wave exposed: Rhinatrematidae (7 of 9), Typhlonectidae (7 of 10), Siphonopidae (11 of 16), Scolecomorphidae (4 of 6), and Caeciliidae (19 of 36). With the exception of Scolecomorphidae (distributed in tropical Africa), these families are all Neotropical. Salamander families had relatively low heat wave exposure. Of these, the most highly exposed was Ambystomatidae (12 of 35 species, 28%), particularly species that occur in Mexico and southwestern United States.

### Droughts

For droughts, during the baseline period, the median value per grid cell was 8 drought events. This increased to a median of 10 events per grid cell during 2010–2019, with 50% of grid cells showing an increase in drought event count. For conversion to a binary drought layer, we used a threshold value of 10, corresponding to the 80th percentile drought event increase (i.e., including only those grid cells experiencing an increase of 10 or more drought events since the baseline period). Drought events increased in several areas with high amphibian diversity, including much of the Amazon basin, the Atlantic Forest of Brazil, Madagascar, southwestern United States, northern Mexico, and continental Europe (Figure 3).

Across amphibians, there were 1143 drought‐exposed species, representing 16% of amphibian species. Caecilians were the most drought‐exposed order (Gymnophiona, 17% of species classified as exposed), followed by frogs (Anura, 16%) and salamanders (Caudata, 10%). For frogs, the highest numbers of drought‐exposed species occurred in Madagascar and the Atlantic Forest (Figure 3). By country, Brazil contained the highest number of drought‐exposed frog species (192 species), followed by Venezuela (164), Madagascar (139), and Colombia (87). For salamanders, Central America, Europe, and southern China were the regions with the highest numbers of drought‐exposed species (Figure 3). Honduras had the highest number of drought‐exposed salamanders (13), followed by China (12), Italy (9), and Spain (8). For caecilians, India had the highest number of drought‐exposed species (16), followed by Venezuela (5), Colombia (4), and Brazil (3). In Madagascar, the proportion of species classified as drought exposed approached 100% along the eastern coast. Similarly, Western Australia, Spain, South Africa, and Chile, all of which harbor relatively large numbers of amphibian species, had high proportions of drought‐exposed species (Figure 5).

There were 6 frog families with at least half the species classified as drought exposed. Rhinodermatidae and Calyptocephalellidae, both from the Southern Cone, with 3 and 5 species, respectively, each had 100% of their species classified as drought exposed. Two other frog families from the same region, Batrachylidae and Alsodidae, also had high proportions of exposed species (Batrachylidae: 7 of 14 species; Alsodidae: 14 of 25 species). Heleophrynidae, containing 6 species from southern Africa, had 5 drought‐exposed species. Mantellidae, endemic to Madagascar, had 108 of 215 species (50%) classified as drought exposed. Among salamanders and caecilian families, drought exposure was low except for the caecilian family Chikilidae, whose lone species was classified as drought exposed (currently, 4 species of Chikilidae are recognized and only one was covered in the IUCN data set at the time of access).

### Cold spells

For cold spells, the median value per grid cell during the baseline period (1980–1989) was 72 cold spell events, whereas in 2010–2019, the median value was 46 events. The median difference was −24 events, indicating that on average there was a reduction in cold spells over time. However, in 6.1% of the grid cells, there was an increase in cold spell events relative to the baseline period. For conversion to a binary layer for further analyses, we included all grid cells with an increase in cold spell events relative to the baseline (i.e., any grid cell with the difference in cold spell events >0), which corresponds to the upper 94th percentile. The cutoff for cold spells differed from the 80th percentile cutoff for heat waves, given that with cold spells the 80th percentile included grid cells where cold spells decreased. Thus, heat waves and cold spells are not directly comparable, and generally speaking cold spell frequency decreased over time. Still, there was an increase in cold spell events in northern Australia, parts of Central Asia, and almost all of southern South America (Southern Cone) (Figure 2).

**FIGURE 4:**
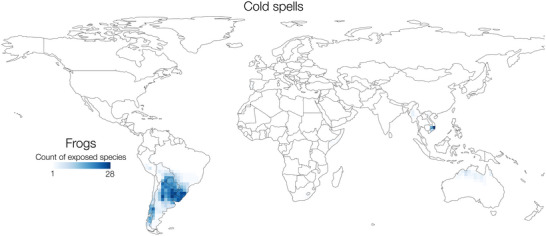
Number of frog species exposed to cold spells per 2° global grid cell. A species is exposed if >50% of its distribution overlaps with the cold spell layer. There are no cold‐spell‐exposed salamander species and 2 exposed caecilian species.

**FIGURE 5:**
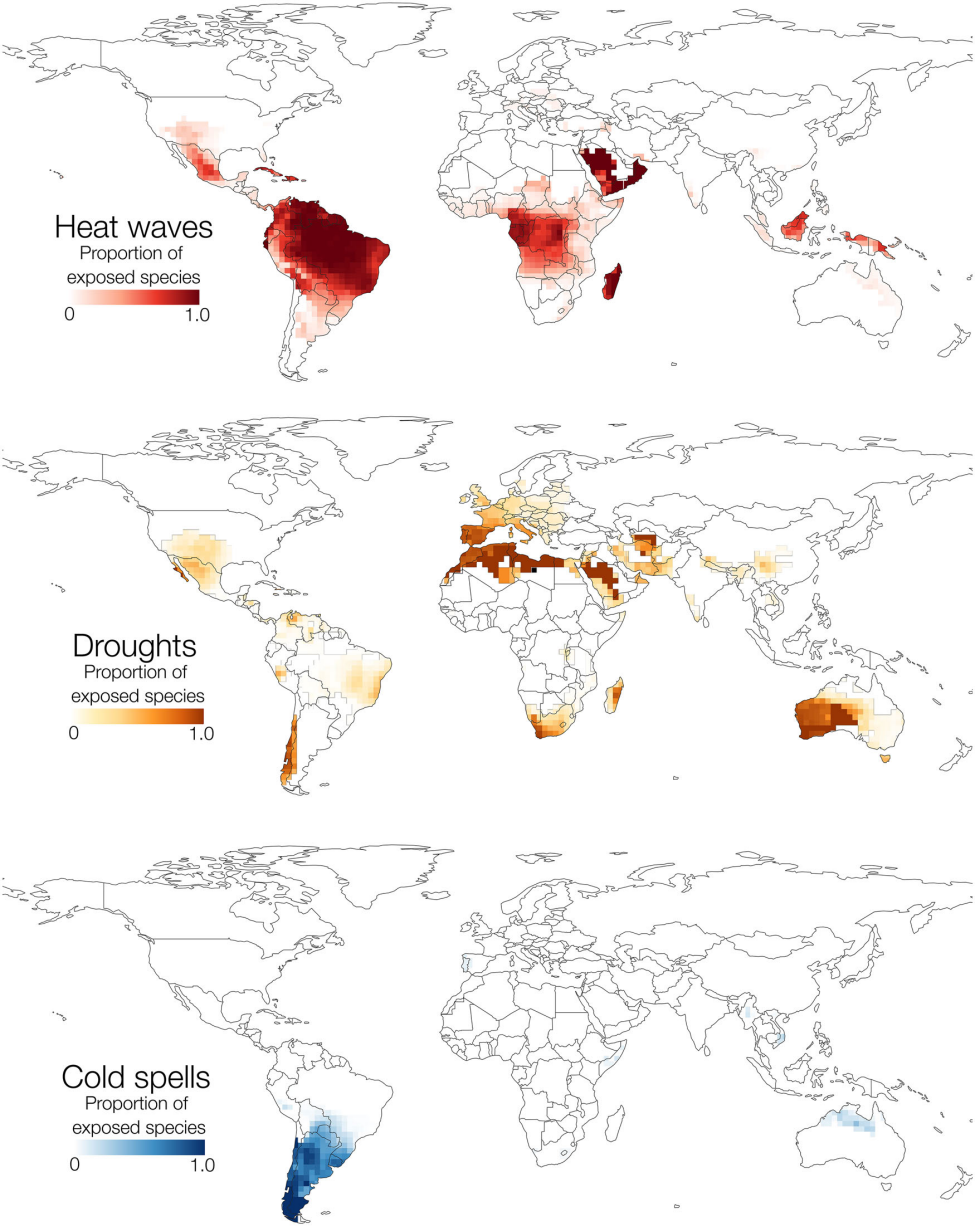
Proportion of amphibian species exposed to each class of extreme events per 2° global grid cell. For each cell, the count of exposed species (Figures 3 & 4) was divided by the total amphibian richness of that cell (Figure 1).

Across amphibians, 199 species (3% of total diversity) were classified as cold spell exposed, all but 2 of which were frogs (the remaining 2 were caecilians). Among frogs, the majority of these cold‐spell‐exposed species occurred in the Southern Cone (Figure 4). Argentina contained, by far, the highest number of cold‐spell‐exposed frog species (101), followed by Brazil (42), Chile, (36), Paraguay (34), Uruguay (30), and Bolivia (30). In these regions, the proportion of exposed amphibian richness was high, reaching nearly 100% in parts of Chile and Argentina (Figure 5).

A number of frog families occurring in the Southern Cone were highly exposed to cold spells, including Batrachylidae (14 of 14 species classified as exposed), Rhinodermatidae (3 of 3 species), Alsodidae (18 of 25 species, 72%), Calyptocephalellidae (3 of 5 species, 60%), and Ceratophryidae (6 of 12 species, 50%).

### Multiple threats

Across all amphibians, 8.6% of species (618 of 7204) were classified as exposed to 2 or more extreme event classes. Of these, the majority were exposed to a combination of heat waves and drought (578 species), which is expected given that the drought index we used, SPEI, accounts for drought severity on the basis of both precipitation and temperature. The combination of cold spell and drought was less common (37 species exposed), and very few species were exposed to both cold spells and heat waves (5 species exposed). Only a single species, *Ameerega boehmei* (Anura, Dendrobatidae), distributed in the Chiquitania region of eastern Bolivia, was classified as exposed to all 3 extreme event classes.

Although all 3 amphibian orders had relatively low proportions of species exposed to multiple extreme events, there were a handful of frog families with high exposure. All 3 species in Rhinodermatidae were classified as exposed to both cold spells and droughts. Similarly, 7 of 14 species in Batrachylidae (50%), 3 of 5 in Calyptocephalellidae (60%), and 11 of 25 in Alsodidae (44%) were exposed to the same combination. All these families occur in the Southern Cone, indicating that the drought and cold spell combination may be a pervasive threat to amphibian species in this region. Half of all species in Mantellidae were heat wave and drought exposed (107 of 215 species, 50%). Aromobatidae and Odontophrynidae, 2 frog families occurring throughout much of South America, also had relatively high exposure to the same combination (Aromobatidae: 43 of 113 species, 38%; Odontophrynidae: 11 of 30 species, 37%).

### Extreme event exposure and changes in IUCN Red List status

Heat wave and drought exposure were positively associated with deteriorating status (being uplisted) in the second reassessment period (2004–2022) but not the first (1980–2004) (Table 1; Appendix S3). Specifically, an increase in heat wave exposure from 0.0 (no exposure) to 1.0 (100% overlap between the species range and the heat wave binary layer) multiplied the odds of being uplisted by 1.84 (*p* < 0.001); for droughts, the odds ratio was slightly lower at 1.66 (*p* = 0.004). Similarly, drought exposure was significantly associated with being downlisted but with an odds ratio <1 (0.18, *p* = 0.006), indicating that a reduction in drought exposure was associated with an increase in the probability of a status improvement (downlisted). During the earlier period (1980–2004), none of the predictors were significantly associated with changes in listing status (Appendix S3).

**TABLE 1:** Results from the multinomial logistic regression analyzing the relationship between exposure to 3 extreme event classes and changes in International Union for Conservation of Nature Red List conservation status from 2004 to 2022.

Outcome^a^	Predictor	Odds ratio	95% CI	*p*
Uplisted	(Intercept)	0.03	0.02–0.03	<0.001
Uplisted	Heat wave exposure	1.84	1.36–2.48	<0.001
Uplisted	Drought exposure	1.66	1.16–2.32	0.004
Uplisted	Cold spell exposure	1.24	0.52–2.94	0.631
Downlisted	(Intercept)	0.02	0.01–0.02	<0.001
Downlisted	Heat wave exposure	0.67	0.37–1.19	0.174
Downlisted	Drought exposure	0.18	0.05–0.61	0.006
Downlisted	Cold spell exposure	0.77	0.16–3.72	0.744

^a^ Uplisted, status deterioration; downlisted, status improvement.

## DISCUSSION

We documented a pattern of increasing extreme events of various forms (heat waves, cold spells, and droughts) and found that amphibian biodiversity is substantially exposed to these events. Furthermore, increased exposure to heat waves and droughts was associated with deteriorating IUCN Red List status from 2004 to 2022 (Table 1). This effect was not seen during the earlier reclassification period (1980–2004) (Appendix S3), suggesting that amphibians may have been exposed to different threats during these two periods. As noted by Luedtke et al. (2023), disease was the dominant driver of status deteriorations from 1980 to 2004, whereas from 2004 to 2022, climate change effects were the most common driver of status deteriorations. Our results suggest that extreme events, namely heat waves and droughts, may be an important causal factor in climate‐related amphibian declines (Table 1).

Of the 3 classes of events we considered, drought is expected to have the most severe consequences on amphibians (Walls et al., 2013). This is because all amphibians depend on fresh water (or environmental moisture) for reproduction and survival, and, in many cases, this is in the form of nonpermanent, seasonal water bodies, such as vernal pools, phytotelmata, and temporary streams. Thus, among the most consequential of drought's effects on amphibians is the premature drying of breeding sites (McMenamin et al., 2008). In addition to catastrophic breeding failure, droughts can result in reduced size at metamorphosis and reduced adult reproductive activity, leading to declines and local extinctions (Daszak et al., 2005; Walls et al., 2013). It is also worth pointing out that a large number of amphibian species (direct developers) do not use standing water at all for breeding and instead rely on environmental moisture and parental care (protecting and hydrating embryos) for reproduction. Across amphibians, 27% of frogs, 32% of caecilians, and 56% of salamanders exhibit direct development (Liedtke et al., 2022). Yet, surprisingly little is known about how this breeding strategy relates to drought susceptibility.

Most direct‐developing amphibians come from humid tropical regions, and it is thought that this evolutionary shift away from aquatic breeding has been facilitated by abundant environmental moisture (Wells, 2007). At least one population of a direct‐developing plethodontid salamander was extirpated due to a short‐term drought (Jaeger, 1980), and a study on direct‐developing frogs in Puerto Rico documented declines coinciding with extended droughts, possibly exacerbated through interactions with Bd (Burrowes et al., 2004). Despite this, some amphibian species demonstrate substantial drought resilience, for example, by phenotypic plasticity in response to unpredictable pond hydroperiods and behavioral flexibility in the timing and location of reproduction (Jakob et al., 2003; Lannoo & Stiles, 2020; Moss et al., 2021; Price et al., 2012). Additional research on the life‐history attributes that influence drought susceptibility and resilience could improve understanding of how amphibians will respond to climate change and extreme events.

We think it is prudent to draw attention to a specific result from our study: drought exposure in European salamanders. Most of Europe was classified as having experienced an increase in drought events, with the exception of the British Isles, Scandinavia, and the Alps (Figure 2). Although our analyses of extreme events are based on prior occurrences, the patterns we found are largely in agreement with future predictions. The IPCC Special Report on extreme events and climate change reports with medium confidence that droughts will increase in duration and intensity in central Europe, the Mediterranean, central North America and Mexico, northeastern Brazil, and southern Africa (Seneviratne et al., 2012). Most European salamanders are members of the family Salamandridae, and we found that approximately half of these (16 species) were classified as exposed. Additionally, 5 European plethodontids in the genus *Speleomantes* were classified as drought exposed. Although several of these salamanders are associated with larger, more permanent wetlands or caves (e.g., *Pleurodeles waltl*), many rely on small ponds and streams both for breeding and as adult habitat, and several are also classified as threatened by the IUCN Red List (e.g., *Salamandrina perspicillata*, *Speleomantes ambrosii*).

Another important result from our study is that so many tropical amphibians were classified as both heat wave and drought exposed. Madagascar, the Amazon, and the Atlantic Forest region of Brazil are of particular concern. Although many of the negative effects of heat waves on amphibians would be presumably through increased water stress (Rollins‐Smith & Le Sage, 2023), heat waves alone could have negative impacts on amphibians, regardless of associated drought. Some effects, such as altered seasonal breeding phenology (Sadinski et al., 2018) and decreased overwintering success (Muths et al., 2020), would be expected to mainly influence temperate amphibians. Other effects, such as decreased immune function, skin microbiome modulation, and interactions with disease, would also be relevant for tropical amphibian species (Rollins‐Smith & Le Sage, 2023).

Although we found that the frequency of cold spells mostly decreased over time, some regions, such as the Southern Cone, experienced a cold spell increase. Cooling trends in the Southern Cone have also been documented in previous studies (Nuñez et al., 2008; Rusticucci, 2012). The effects of cold spells on amphibians are still somewhat unclear, but there is evidence to suggest that Bd is more severe during cold periods (Bradley et al., 2019; Campbell et al., 2019; Drew et al., 2006; Raffel et al., 2013). Many of the amphibian species in the regions where cold spells increased are also highly susceptible to Bd. There were 5 families of frogs for which half or more of the species were classified as cold spell exposed, all of which occur in the Southern Cone. Reviewing global patterns of Bd prevalence, Olson et al. (2021) found that all 5 of these families also had high Bd prevalence. For example, the family Batrachylidae contains 14 species (in the IUCN data set, 12 were represented at the time of this writing), all of which were classified as cold spell exposed. Six of the 7 species that have been tested for Bd have tested positive (Olson et al., 2021). Similar results were found in mouth‐brooding frogs of the family Rhinodermatidae (100% cold spell exposed, 100% Bd prevalence), and it is suspected that Bd may be driving both species of *Rhinoderma* to extinction (Soto‐Azat et al., 2013). Although there are anecdotal reports of Bd‐related declines occurring during cold periods (e.g., 2021–2022 winter frog die‐offs in Australia), more research is needed to determine the links between these two stressors and whether they contribute synergistically to amphibian declines.

What kind of management strategies might buffer the effects of extreme events on amphibians? Shoo et al. (2011) reviewed this topic with respect to climate change (not necessarily focused on extreme events) and argued that the installation of microhabitat refuges and amelioration of wetland habitats (e.g., manipulating hydroperiod) would be worthwhile strategies. Several of the proposed actions would be beneficial in tempering the effects of heat waves and droughts. As discussed above, a significant fraction of amphibian species do not require standing water for reproduction (e.g., direct developers) but do require humid microhabitats. Thus, providing humid refuges (e.g., PVC pipes, cover boards) and modifying microhabitats may be an effective way to buffer the effects of certain extreme events. Additionally, targeted amelioration of the effects of droughts via artificial irrigation may be warranted in certain cases (e.g., the Kihansi spray toad [Weldon et al., 2020]). Other actions, such as excavation of small ponds, protection of narrow zones of riparian habitat, and replenishing wetlands with groundwater (Shoo et al., 2011), may all be useful strategies for mitigating the effects of climate change and extreme events on amphibians.

Earth's ecosystems face a growing threat from a combination of significant stressors (IPBES, 2019). Although climate change has emerged as a leading threat to amphibians in the past 2 decades, other global drivers of amphibian biodiversity loss have also been at play (Luedtke et al., 2023). These drivers include various forms of habitat degradation, chemical pollution, overcollection, the spread of diseases, and the introduction of invasive species (Luedtke et al., 2023). Although relatively simple habitat restoration measures have proven effective in certain cases (Moor et al., 2022), it is becoming increasingly clear that, as with other taxonomic groups and ecosystem functions (Albacete et al., 2023; Lemm et al., 2021), multiple stressors often combine to erode amphibian populations and biodiversity. Extreme events are powerful drivers shaping amphibian populations that can be examined in conjunction with other growing stressors (Côté et al., 2016) to enhance our predictions for the future.

## Supporting information



Appendix S1. Results of the calculation of the extreme events layers using different percentile thresholds for the differences in counts of events between recent and baseline time periods.
Appendix S2 (attached as a separate file). Exposure of amphibians to heat waves, cold spells, and droughts, aggregated to different taxonomic levels (species, order, family, and genus).
Appendix S3. Results from the multinomial logistic regression analyzing the relationship between exposure to three extreme event classes and changes in International Union for Conservation of Nature Red List conservation status from 1980 to 2004.

Supplementary Information
