# Supplementary material for: Quantifying exposure of amphibian species to heat waves, cold spells, and droughts
Source: Conserv Biol. 2025 May 31;39(5):e70074. doi: 10.1111/cobi.70074 (PMC12451495; doi:10.1111/cobi.70074)
Supplement: Supplementary file 1 — Appendix S1. Results of the calculation of the extreme events layers using different percentile thresholds for the differences in counts of events between recent and baseline time periods. Appendix S2 (attached as a separate file). Exposure of amphibians to heat waves, cold spells, and droughts, aggregated to different taxonomic levels (species, order, family, and genus). Appendix S3. Results from the multinomial logistic regression analyzing the relationship between exposure to three extreme event classes and changes in International Union for Conservation of Nature Red List conservation status from 1980 to 2004. [file COBI-39-e70074-s002.docx]

Appendix


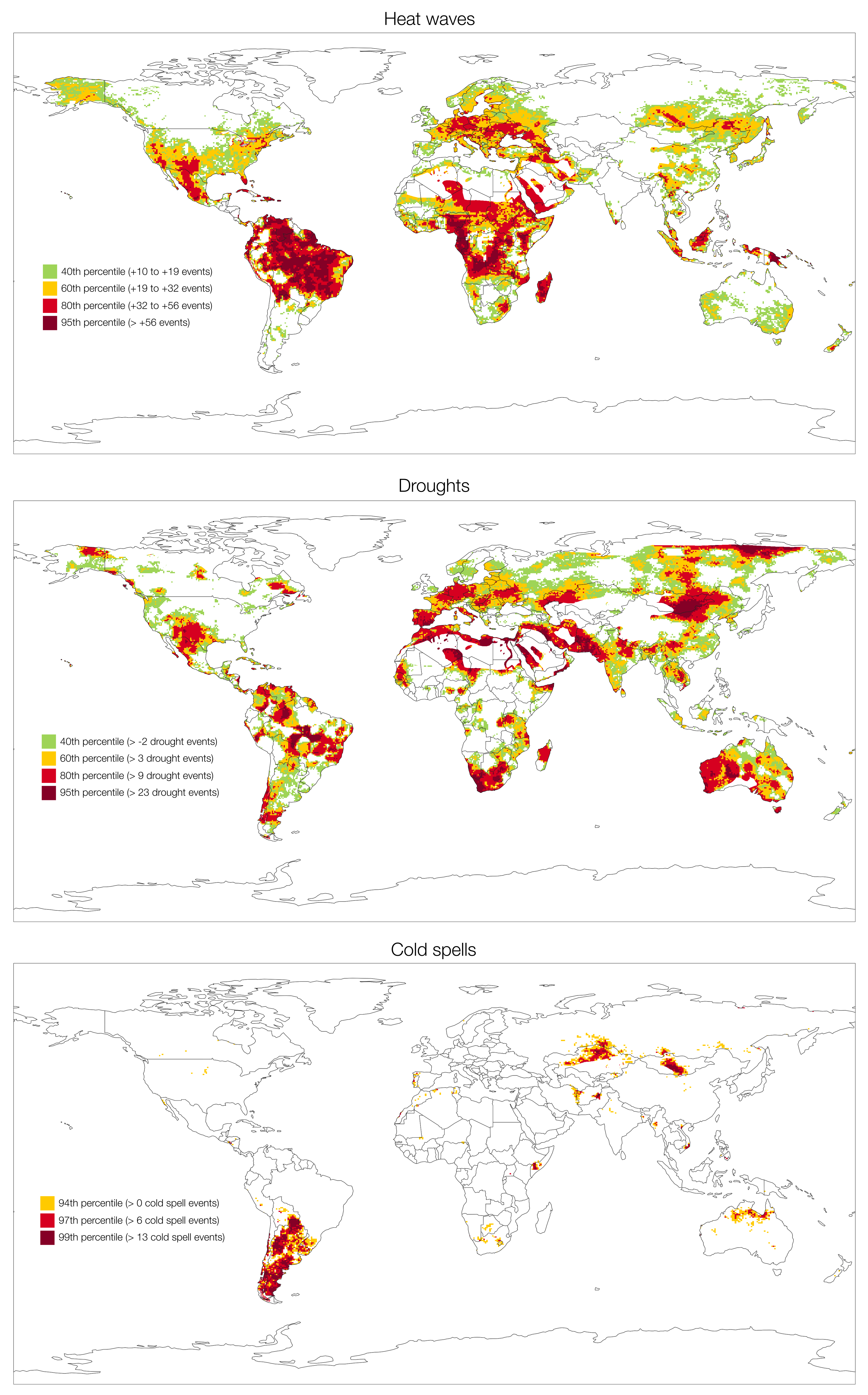


**Appendix S1.** Results of the calculation of the extreme events layers using different percentile thresholds for the differences in counts of events between recent and baseline time periods.

**Appendix S2** (attached as a separate file). Exposure of amphibians to heat waves, cold spells, and droughts, aggregated to different taxonomic levels (species, order, family, and genus).

**Appendix S3.** Results from the multinomial logistic regression analyzing the relationship between exposure to three extreme event classes and changes in International Union for Conservation of Nature Red List conservation status from 1980 to 2004.

| **Outcome*** | **Predictor** | **Odds ratio** | **95% CI** | ***p*** |
| --- | --- | --- | --- | --- |
| uplisted | (Intercept) | 0.07 | 0.06–0.08 | < 0.001 |
| uplisted | Heat wave exposure | 1.02 | 0.80–1.29 | 0.893 |
| uplisted | Drought exposure | 0.88 | 0.64–1.21 | 0.444 |
| uplisted | Cold spell exposure | 0.88 | 0.44–1.75 | 0.709 |
| downlisted | (Intercept) | 0.01 | 0.00–0.01 | < 0.001 |
| downlisted | Heat wave exposure | 0.64 | 0.24–1.72 | 0.379 |
| downlisted | Drought exposure | 0.86 | 0.25–2.96 | 0.809 |
| downlisted | Cold spell exposure | 2.36 | 0.43–12.80 | 0.321 |

*Uplisted indicates a status deterioration, downlisted indicates status improvement.
